# Postreconstruction filtering of 3D PET images by using weighted higher-order singular value decomposition

**DOI:** 10.1186/s12938-016-0221-y

**Published:** 2016-08-27

**Authors:** Hongbo Liu, Kun Wang, Jie Tian

**Affiliations:** 1 Engineering Research Center of Molecular and Neuro Imaging of the Ministry of Education and School of Life Science and Technology, Xidian University, 266 Xinglong Section of Xifeng Road, Xi’an, 710126 China; 2 Key Laboratory of Molecular Imaging, Institute of Automation, Chinese Academy of Sciences, 95 Zhongguancun East Road, Beijing, 100190 China

**Keywords:** Positron emission tomography (PET), Higher-order singular value decomposition (HOSVD), Anscombe variance-stabilizing transform (AVST), Similarity, Weighted, Denoising

## Abstract

**Background:**

Positron emission tomography (PET) always suffers from high levels of noise due to the constraints of the injected dose and acquisition time, especially in the studies of dynamic PET imaging. To improve the quality of PET image, several approaches have been introduced to suppress noise. However, traditional filters often blur the image edges, or erase small detail, or rely on multiple parameters. In order to solve such problems, nonlocal denoising methods have been adapted to denoise PET images.

**Methods:**

In this paper, we propose to use the weighted higher-order singular value decomposition for PET image denoising. We first modeled the noise in the PET image as Poisson distribution. Then, we transformed the noise to an additive Gaussian noise by use of the anscombe root transformation. Finally, we denoised the transformed image using the proposed higher-order singular value decomposition (HOSVD)-based algorithms. The denoised results were compared with results from some general filters by performing physical phantom and mice studies.

**Results:**

Compared to other commonly used filters, HOSVD-based denoising algorithms can preserve boundaries and quantitative accuracy better. The spatial resolution and the low activity features in PET image also can be preserved by use of HOSVD-based methods. Comparing with the standard HOSVD-based algorithm, the proposed weighted HOSVD algorithm can suppress the stair-step artifact, and the time-consumption is about half of that needed by the Wiener-augmented HOSVD algorithm.

**Conclusions:**

The proposed weighted HOSVD denoising algorithm can suppress noise while better preserving of boundary and quantity in PET images.

**Electronic supplementary material:**

The online version of this article (doi:10.1186/s12938-016-0221-y) contains supplementary material, which is available to authorized users.

## Background

Positron emission tomography (PET) is a non-invasive high-specificity imaging modality that is widely used for both clinical diagnosis and preclinical research because of its ability to reveal the metabolic evolution of molecular and biochemical processes in living organs. However, it suffers from relatively poor spatial resolution and high levels of noise due to the constraints of the injected dose and acquisition time, especially in the studies of dynamic PET imaging. High noise levels make the quantitative interpretation of PET images difficult; this is especially problematic when the image is to be used for early tumor detection when the tumor is small [1], myocardial perfusion [2], or hypometabolism of glucose in studies of early diagnosis of Alzheimer’s disease [3, 4]. In such applications, high noise level contained in PET images will cover small but important lesions and make the edges between organs blurred, further leading to mistakes in diagnosis and quantitation. Therefore, noise reduction is important for interpretation and computer-aided analysis of PET images.

Several approaches have been introduced to suppress noise contained in PET images, during or after image reconstruction process. Statistical image reconstruction method such as maximum likelihood expectation maximization (MLEM) [5], its accelerated version ordered subset expectation maximization (OSEM) [6], or other modified algorithms [7–10] can model the noise in acquired data but suffer from increased noise as iterations increase. The regularization algorithm [11, 12], which reduces noise in the statistical reconstruction process by the way of adding a prior or penalty term to the reconstruction process, has promised a good denoising performance, but the determination of the appropriate regularization parameters is a challenging work, which is usually task-dependent. Alternatively, a smoothing filter [11, 13] after reconstruction, such as a Gaussian filter (GF), is widely used in current clinical applications. This kind of filter suppresses noise by averaging adjacent pixels by using a weight function of spatial distance between pixels [14], and has an inherent drawback of blurring edges, which may be clinically relevant. In [15], the authors used masked smoothing method for CT perfusion images denoising, but its performance for PET images has not been proved.

Numerous edge-preserving image filters, such as the bilateral filter (BF) [16, 17], anisotropic diffusion filters (ADFs) [18, 19], or priors [11, 20], which are able to detect and maintain edges while smoothing over noise-induced fluctuations, have been introduced to denoise PET images. However, these approaches rely on multiple parameters specified by users according to the study to control the smoothness level and target intensity levels. Actually, it is impossible to preserve all of the interesting features simultaneously [21, 22]. Moreover, these filters usually erase small details, such as small tumors, which can lead to misdiagnosis. Algorithms that incorporate the anatomical information from CT or MRI into PET reconstruction [19, 23, 24] will improve the denoising performance, but the improvement of performance can only be achieved when the boundaries from anatomical image and PET image coincide with each other strongly; if not, these algorithms may introduce incorrect information leading to bias and artifacts [25]. Transform-based filters, such as wavelet-based filters [26–28] and singular value decomposition [29], have also been proposed to denoise PET images by exploiting the uncorrelated properties of noise in the transform domain.

Recently, nonlocal denoising methods, such as the nonlocal mean (NLM) filters [30] and block-matching 3D (BM3D) filter [31], have been adapted to denoise medical images [32–35], which exploit the similarity and sparsity among small patches to preserve details while removing noise, and achieve outstanding performance. In [21], the authors extended the application of the NLM filter to the 3D PET image and modified it by incorporating anatomical information from the CT image to constrain the similarity measure within a subset of voxels. This algorithm can produce improved lesion contrast and signal-to-noise ratio (SNR) when compared with other traditional PET image denoising methods.

The higher-order singular value decomposition (HOSVD) [36] is another kind of nonlocal denoising method, which is a development on high-order matrix or tensor of the singular value decomposition (SVD) of the two-dimension matrix. It offers a simple method for handling sparsity among similar patches by grouping them into a high-order matrix, then denoising the image in a transform-threshold-inverse transform fashion. The transform bases are learned from the original image and are more adaptable to the image content and may achieve a much sparser representation than fixed bases used in the BM3D method.

The HOSVD-based method has been extended into the applications of MR image denoising, and demonstrated state-of-the-art performance [37]. In this study, we aim to investigate and extend the application of HOSVD to denoise 3D PET images. We examined its ability to preserve high-intensity features in denoised images, and the ability to detect potentially low remnant activity by the percentage recovered signal (PRS) in lesion volume of interest (VOI) and the contrast-to-noise ratio (CNR) in the denoised images. Results were compared to those obtained after GF, BF, and ADF filtering of the same data.

## Methods

To extend HOSVD to PET image denoising and propose our HOSVD-based 3D PET images denoising method, we will first give a brief introduction about HOSVD in image denoising. Then, we will introduce the Anscombe variance-stabilizing transformation and its exact unbiased inverse. Finally, we will describe our method and its implementation.

### Non-local HOSVD denoising for 2D images

Similar to most denoising algorithms, the HOSVD denoising method is originally designed for the additive zero mean Gaussian noise. The main assumption in the denoising process is that the observed noisy image *Y* from a noise free image *X* with additive Gaussian noise *N* can be modeled as $Y = X+N$, where *N* has a known variance ${\sigma }^2$ and zero mean.

To find a good estimate $\tilde{X}$ of *X* from *Y*, the HOSVD denoising method first groups the similar patches for each reference image patch into a 3D stack. Then, the HOSVD transformation of the stack is performed to obtain its representation by the HOSVD bases and coefficients. Based on the uncorrelated properties of noise in the HOSVD transform domain, the coefficients are truncated by a hard thresholding operator, then one denoised estimate of this stack is obtained by the inverse HOSVD transform. Repeating this operation for each reference patch results in multiple estimates for each pixel. The final denoised image is obtained by averaging these estimates. The details of the standard HOSVD denoising process are described in the following.

Given a $p\,{\times }\,p$ reference patch $P_{ref}$ in the original noisy 2D image, *K* similar patches are found including $P_{ref}$ and stacked into a 3D matrix $Z\,{\in}\,R^{p\,{\times }\,p{\times }\,K}$. Then, the HOSVD of *Z* is performed and can be formulated as [38]

1 $$\begin{aligned} Z = S\,{\times }\,_{1}U^{(1)}\,{\times }\,_{2}U^{(2)}\,{\times }\,_{3}U^{(3)}, \end{aligned}$$

where $U^{(1)},U^{(2)}\,{\in}\,R^{p\,{\times }\,p}$, and $U^{(3)}\,{\in}\,R^{K\,{\times }\,K}$ are orthonormal unitary matrices. They are computed from the SVD of the unfolding matrices of *Z* along three directions respectively [38]. ${\times }_n$ stands for the *n*-mode tensor product. *S* is a 3D coefficient matrix of size $p\,{\times}\,p\,{\times}\,K$. Eq. (1) indicates that the HOSVD can exploit signal sparsity across each dimension.

The coefficient matrix *S* can be computed as:

2 $$\begin{aligned} S = Z\,{\times }\,_{3}{U^{(3)}}^{T}\,{\times }\,_{2}{U^{(2)}}^{T}\,{\times }\,_{1}{U^{(1)}}^{T}, \end{aligned}$$

where *T* represents the transpose of a matrix. Then, stack *Z* can be filtered by a hard-threshold operation for *S*, and the threshold $\tau$ can be determined as follows [39]:

3 $$\begin{aligned} \tau =\sigma \sqrt{2\log (p^2K)}. \end{aligned}$$

Thus, the truncated coefficient matrix $\tilde{S}$ with entries $\tilde{s}_i$ can be determined as follows:

4 $$\begin{aligned} {\tilde{s}_i} = \left\{ { \begin{array}{*{20}{c}} {{s_i}}&{}\quad{\mathrm{{for}}\, \left| {{s_i}} \right| \ge \tau }\\ 0&{}\quad{\mathrm{{for}}\, \left| {{s_i}} \right| < \tau } \end{array}} \right. \end{aligned}$$

where the threshold $\tau$ is optimal from a statistical risk viewpoint for any orthonormal basis and for $N{\sim }\mathcal {N}(0,\sigma ^2)$. This truncation of the HOSVD coefficients is based on the assumption that the underlying noise-free image can be sparsely represented by the HOSVD bases. Using (1), we can obtain an estimate $\tilde{Z}$ of *Z* by following:

5 $$\begin{aligned} \tilde{Z} = \tilde{S}\,{\times }\,_1{U^{(1)}}\,{\times }\,_2{U^{(2)}}\,{\times }\,_3{U^{(3)}}. \end{aligned}$$

Repeating the above process for each reference patch in sliding window fashion, multiple estimates at each pixel will be obtained. The final filtered image is produced by averaging these estimates at each pixel.

To improve the denoising performance even further, an additional Wiener filter step after the standard HOSVD denoising process can be performed. Let $\tilde{Z}$ be a stack of similar patches from the standard HOSVD filtered image, which corresponds to *Z* from the original noisy image, then the coefficients of $\tilde{Z}$ and *Z* in the HOSVD bases of $\tilde{Z}$ are denoted as $\tilde{s}$ and *s* respectively. Then, the filtered coefficients of *Z*, denoted as $\bar{s}$, are computed as follows:

6 $$\begin{aligned} \bar{s} = \frac{s{\tilde{s}}^2}{{{\tilde{s}}^2}{+}{{\sigma }^2}}. \end{aligned}$$

In this paper, the standard HOSVD and its Wiener filter-augmented version are referred to as HOSVD-S and HOSVD-W respectively.

### HOSVD-based denoising for 2D PET images

The above-mentioned HOSVD denoising for 2D images cannot be straightly extended to denoise PET images, as (1) for nonlinear reconstruction algorithms, usually, the exact noise model in reconstructed PET images is unknown, even though raw PET data follow the Poisson distribution, and (2) the intensity value at each voxel in PET images is the representation of the activity concentration (Bq/ml) in the corresponding region of the space, which is low, so the assumption of an image-independent noise model does not hold.

In fact, for images with low photon counts, such as a PET image or SPECT image, the pixel or voxel intensity can be modeled approximatively with the Poisson distribution, which follows the observations that the noise variation in PET images are multiplicative, asymmetric and varying in the image. In this paper, we approximate noise in reconstructed PET images with the Poisson model. Then, with the Anscombe root transformation [40] and its inverse, the HOSVD-based denoising algorithms can be used to denoise PET images. Here, we employ the closed-form approximation of the exact unbiased inverse of the Anscombe variance-stabilizing transformation [41] to alleviate the biased estimates caused by the nonlinearity of the Anscombe transformation. The Anscombe transformation and its inverse are denoted as $AVST$ and $IAVST$, respectively, which are formulated as follows:

7 $$\begin{aligned} AVST (I)=2\sqrt{I{+}3/8} \end{aligned}$$

and

8 $$\begin{aligned} IAVST (Z) = \frac{1}{4}Z^2+\frac{1}{4}\sqrt{\frac{3}{2}}Z^{-1}-\frac{11}{8}Z^{-2}+\frac{5}{8}\sqrt{\frac{5}{8}}Z^{-3}-\frac{1}{8}, \end{aligned}$$

where *I* represents the noisy PET image, and *Z* is the denoised data in the HOSVD transform domain.

The HOSVD-based denoising algorithms can then be extended to the denoising application of PET images by using the following formula:

9 $$\begin{aligned} \tilde{I}= IAVST ({Den}_{\scriptscriptstyle {HOSVD}}( AVST (I))), \end{aligned}$$

where ${Den}_{\scriptscriptstyle {HOSVD}}(\cdot )$ represents the HOSVD denoising manipulation, *I* and $\tilde{I}$ are the noisy and denoised PET images, respectively.

### Weighted HOSVD denoising scheme for 3D PET images

**Fig. 1:**
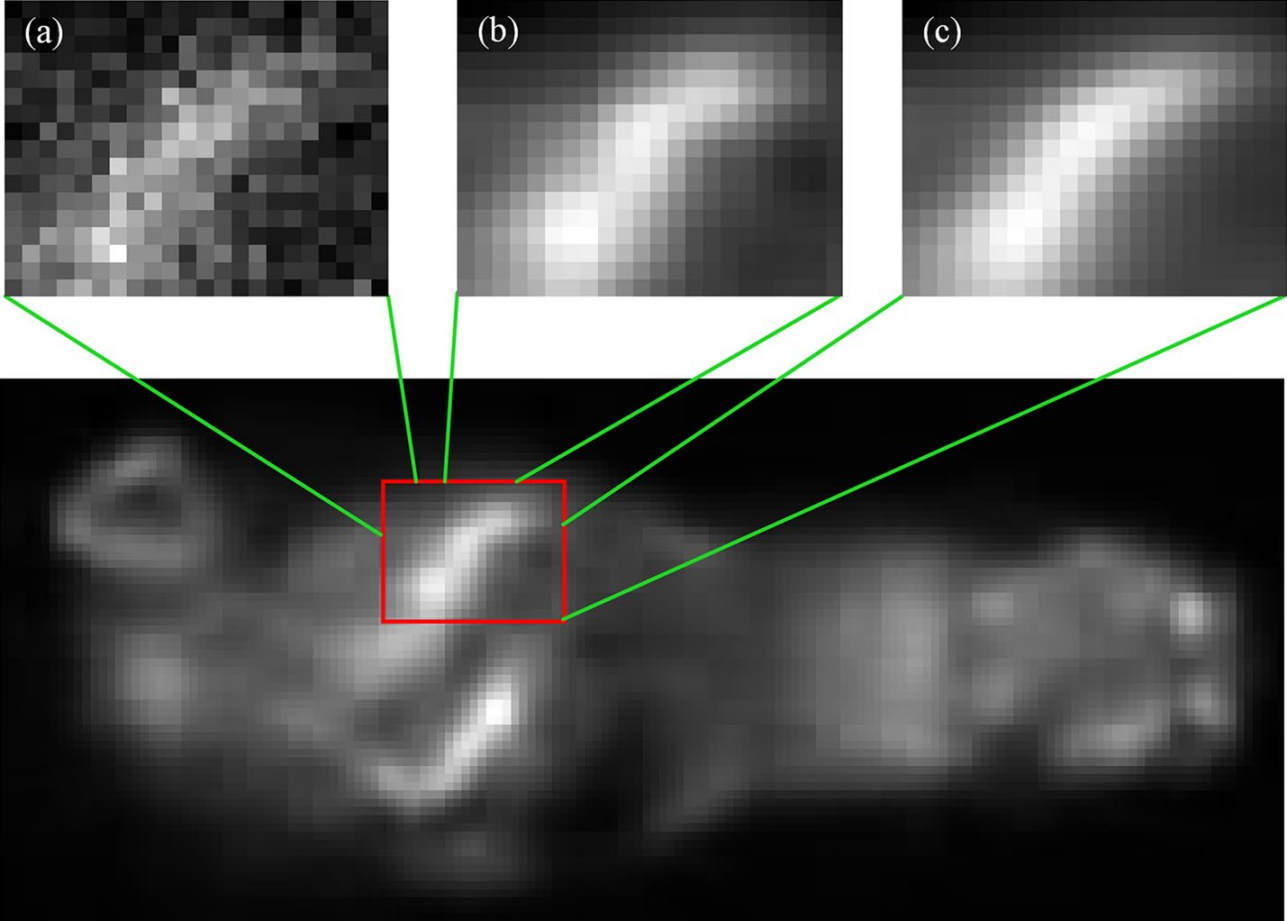
An example denoised image resulting from the straight usage of HOSVD-S in 3D PET image. We given the zoom of a local region of interest in *b*, as a contrast, we also shown the zoom of corresponding regions in original image *a* and the denoised image with method proposed in this study *c*

The hard-threshold operation used in HOSVD-S may result quite different between reference and selected similar patches in structure; this is especially true when the exact structure is unavailable in the underlying clean images, as with PET image denoising. In such applications, straight use of HOSVD-S may lead to stair-step artifact in the denoised image, as shown in Fig. 1. Although the HOSVD-W can modify this disadvantage, its use also increases time consumption dramatically to about twice the time required for HOSVD-S.

To improve the denoising performance of the HOSVD while keeping the time consumption from dramatically increasing, we assign different weights to different selected similar patches, depending on their level of similarity with the reference patch, in structure and statistics. This is different to the HOSVD-S method, which assign identical weight to each selected similar patch. The similarity level in statistics is measured by the *p*-value output of the Kolmogorov-Smirnov (K-S) test between reference patch and the selected similar patch.

For the calculation of weights, we first consider the patch similarity measure used in the HOSVD-S. The distance between the three-dimension (3D) reference patch $P_{ref}$ and its *i*th similar patch $P_i$ can be formulated as:

10 $$\begin{aligned} d(P_{ref},P_i) = \sqrt{{\Vert P_{ref}-P_i\Vert }^2}. \end{aligned}$$

Note that the reference patch $P_{ref}$ and a patch *P* within the search window is similar only if the Euclidean distance $d(P_{ref},P)$ between them meets the following rule [36]:

11 $$\begin{aligned} d^2(P_{ref},P)<3{\sigma }^2p^3. \end{aligned}$$

Based on this, we calculate the similarity weight between $P_i$ and $P_{ref}$ as follows:

12 $$\begin{aligned} s(P_{ref},P_i) = \exp \left(-\frac{1}{2}d^2(P_{ref},P_i)\right), \end{aligned}$$

which means that patches with high similarity to the reference patch will be assigned a larger weight.

To alleviate the disadvantage caused by the original similarity measure, we used a hypothesis test (the one-sided Kolmogorov-Smirnov test) to check how well the values in $P_{ref}-P_i$ conform to $\mathcal {N}(0,2\sigma ^2)$, and the *p*-value output by the K-S tests are used to modify the weighting factor by a multiplication factor. We denote the *p* value output by the K-S test as $p_{KS}(P_{ref},P_i)$, then the weight of patches that are similar to the reference patch $P_{ref}$ can be formulated as follows:

13 $$\begin{aligned} c(P_{ref},P_i) = s(P_{ref},P_i){\cdot }p_{KS}(P_{ref},P_i). \end{aligned}$$

Thus, the *i*th 3D patch that is similar to $P_{ref}$ can be formulated as follows:

14 $$\begin{aligned} \hat{P}_{i} = c(P_{ref},P_i)P_{i}. \end{aligned}$$

Then, the clustering 4D stack can be formulated as:

15 $$\begin{aligned} C(P_{ref}) = (P_{ref},\hat{P}_{1},\hat{P}_{2},...). \end{aligned}$$

Note that $C(P_{ref})$ is a matrix of order four. The denoised 4D stack is formulated as:

16 $$\begin{aligned} \tilde{C}(P_{ref}) = Den_{\scriptscriptstyle {HOSVD}}(C(P_{ref})) = (\tilde{P}_{ref},\tilde{P}_{1},\tilde{P}_{2},...). \end{aligned}$$

The estimation $\breve{P}_i$ of $P_i$ can then be obtained as follows:

17 $$\begin{aligned} \breve{P}_i = \frac{1}{c(P_{ref},P_i)}\tilde{P}_i. \end{aligned}$$

**Fig. 2:**
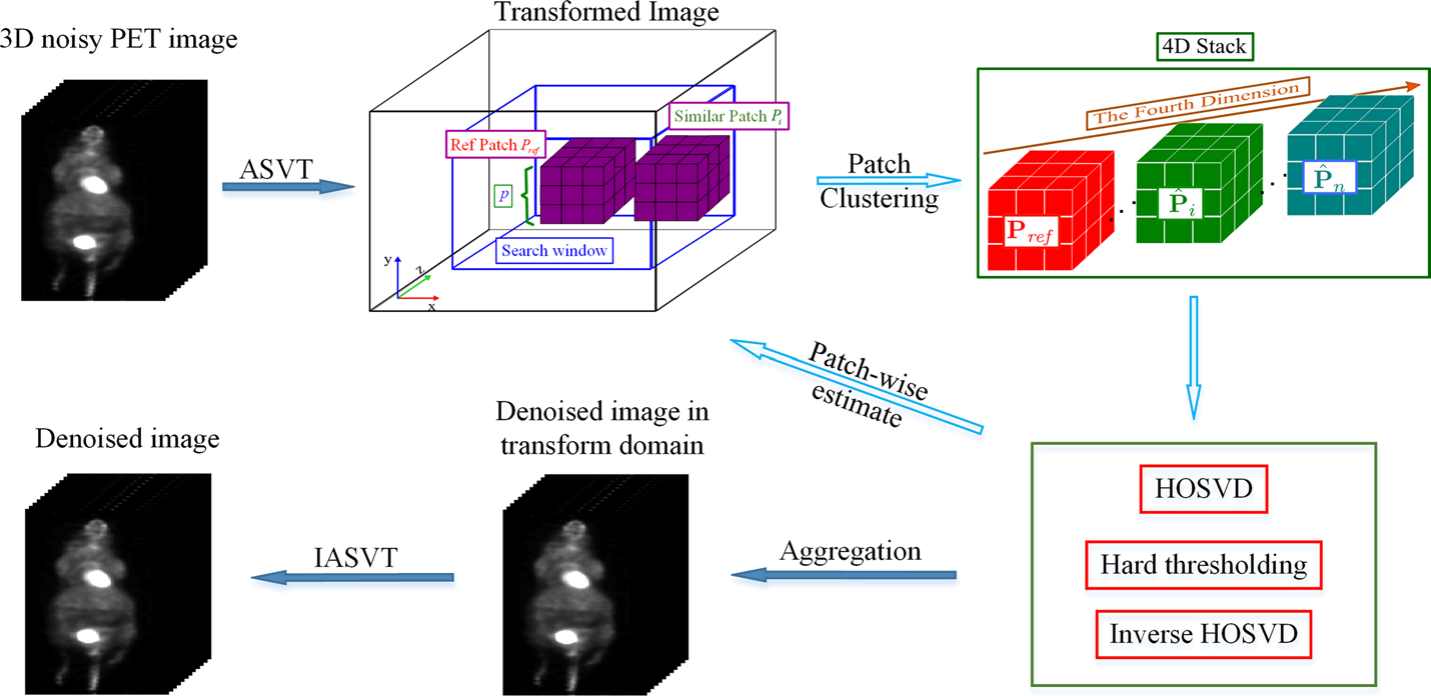
Flow diagram of the proposed HOSVD-KSW algorithm. Note that, the reference patches were obtained by certain voxels for saving execute time. A 4D stack was formed for every reference patch, by clustering itself and its similar patches, with a weighted manner. The stack was then processed orderly by HOSVD, truncated transformation coefficients, and inverse HOSVD, to obtain a estimation value of voxels included in the stack

Repeating the above process for each reference patch and averaging the estimates at each voxel, we can obtain the denoised image in the Anscombe transform domain. The final filtered PET image can then be produced by performing the $IAVST (\cdot )$ operation. For clarity, the proposed weighted HOSVD denoising algorithm is referred to as HOSVD-KSW, and the flow diagram is shown in Fig. 2.

HOSVD-KSW not only preserves all the advantages of the HOSVD-S method, but suppresses the stair-step artifact caused by the HOSVD-S with similar time consumption.

### Practical implementation of the HOSVD-based denoising algorithm

In this work, three HOSVD-based methods are applied to denoise 3D PET images. The most time-consuming parts of HOSVD-based algorithms are 3D patch grouping and HOSVD transform. In the HOSVD-S and HOSVD-W implementation, the K-nearest-neighbors of the reference patch are found based on (11) and are then grouped into a stack. For HOSVD-KSW, the patches that correspond to the first K-maximum-weight are selected as the K-nearest-neighbors of the reference patch (including the reference patch itself) and similar patches obtained by using (11).

To improve the computation efficiency while preserving the denoising efficacy of algorithms, we restricted the size of the searching space of similar patches and the sliding step of the reference patch. On one hand, bigger search windows do not always lead to better results, and a further increase of the search window will increase time consumption, especially in 3D PET image denoising, where similar structures of a special reference patch are usually concentrated in a restricted area close to it. On the other hand, the algorithm execution time is linearly corrected with the number of reference patches selected in the implementation.

In our implementation of the HOSVD-based algorithms, the size of the search window is set to 20 voxels and the sliding step is set to $p-1$ at each image dimension, where *p* is the size of the reference patch in each dimension.

## Experiments and results

### Phantom study

To assess various effects in image quality resulting from different denoising methods, two physical phantom studies were performed, one for the mini-Derenzo phantom and another for the small animal image quality phantom which is described by NEMA NU 4-2008 [42].

**Fig. 3:**
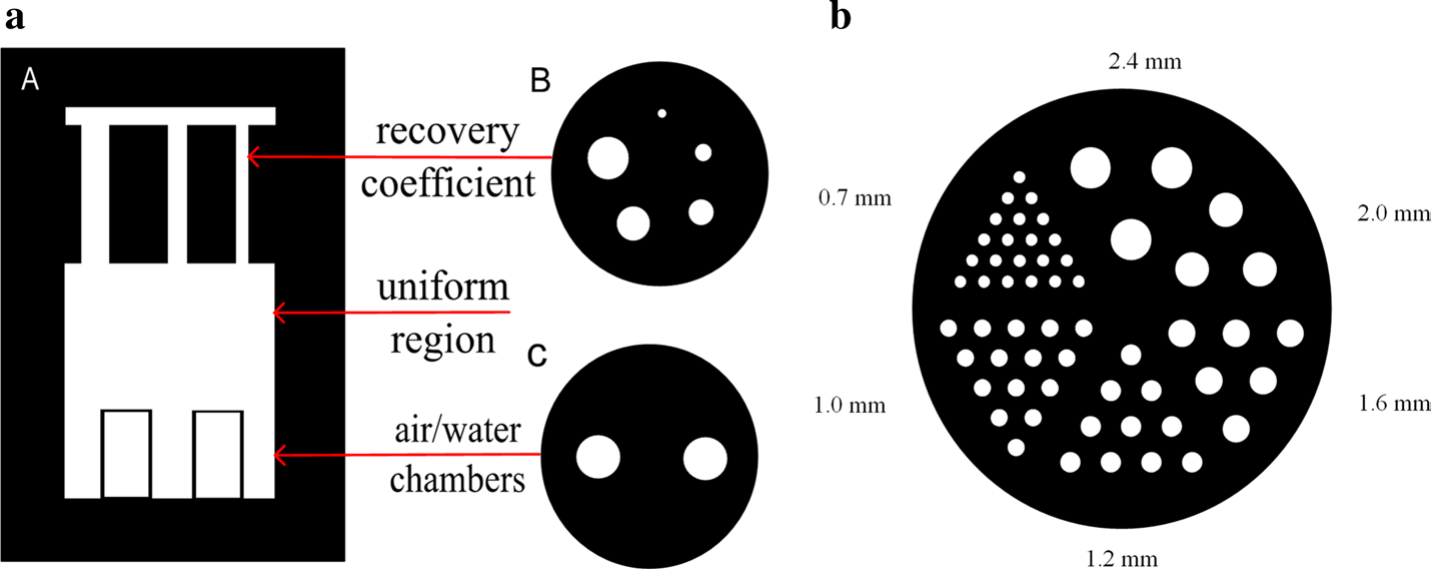
Physical phantom. The NEMA small animal image quality phantom (**a**) contains five rods (*B*) (diameter 1–5 mm) was used to measure the recovery coefficients, homogeneous region to measure the uniformity (*A* middle), and 2-chamber region (*C*) (water- and air-filled) to measure the spill-over ratio. The mini-Derenzo phantom (**b**) was used to measure the effects of different filters on the spatial resolution of PET images

The NEMA small animal image quality phantom (homemade), created from polymethylmethacrylate, has three main parts, as shown in Fig. 3a. The first is a homogeneous cavity with a 30 mm diameter and 30 mm length, filled with radioactivity (hot region) to measure the uniformity. The second part consists of two chambers housed in the cavity and filled with water and air (cold regions, 15 mm in length, wall thickness and outer diameter are 1 and 10 mm respectively) to estimate the spill-over ratio. The third part of the phantom contains five rods which are drilled through a plastic region and have diameters of 1–5 mm respectively, and are used to measure the noise and recovery coefficients.

The mini-Derenzo phantom with rod diameters ranging from 0.7 to 2.4 mm was used to compare the spatial resolution characteristics of the different methods. Fig. 3b is the schematic diagram of the mini-Derenzo phantom.

The small animal image quality phantom was filled with 2.74 MBq of 18F-FDG, and the mini-Derenzo phantom had 1.11 MBq. Data were acquired for 15 min for both phantoms using a GENISYS$^4$ PET scanner (Sofie Biosciences Inc., USA). The images were reconstructed on the GENISYS$^4$ Acquisition Engine with 3D OSEM algorithm (without PSF) into 96 × 96 × 208 voxels, where the voxel dimensions were 0.457 × 0.457 × 0.457 mm.

### Nude mice 18F-FDG study

To compare the different denoising approaches in terms of their effect on preclinical or clinical quantitative studies, we performed an 18F-FDG PET study of nude mice with 4T1 tumors. A 2.24 MBq injection of 18F-FDG mixed with isotonic saline to a total volume of 150 $\upmu$L was administered into nude mice through a tail vein with the mouse immobilized in a restrainer. Image acquisition started 45 min after injection and was performed on the GENISYS$^4$ PET scanner without attenuation correction.

### Quantitative evaluations

#### Uniformity

To quantitatively evaluate and compare the effect of different denoising algorithms on the uniformity of the PET image, a 22.5 mm diameter (75 % of the active diameter) by 10 mm long cylindrical volume of interest (VOI) was drawn over the center of the uniform region of the NEMA small animal image quality phantom. The average activity concentration in this VOI, and its percentage standard deviation (%*STD*) were calculated. The percentage standard deviation was calculated as follows:

18 $$\begin{aligned} \% STD\,=\,100\,\times \,\frac{ STD _{uniform}}{ Mean _{uniform}}, \end{aligned}$$

where

19 $$\begin{aligned} STD _{uniform}\,=\,\sqrt{\frac{1}{n-1}\sum \limits _{i \in VOI }(Act_i- Mean _{uniform})^2} \end{aligned}$$

is the standard derivation of activity concentration in a uniform region VOI, and ${Act}_i$ is the activity concentration in the *i*th voxel of the VOI, and *n* represents the number of voxels that are contained in uniform region VOI. $STD_{uniform}$ and $Mean_{uniform}$ are the standard deviation (STD) and mean in the uniform region VOI, respectively.

#### Recovery coefficients

A single image slice with lower noise characteristics was obtained by averaging image slices that cover the central 10 mm length of the rods. Circular region of interests (ROIs) were taken in this image, around each rod with diameters twice the physical diameter of the rods [42]. Transverse image pixel coordinates of the locations with the maximum voxel value within each of these ROIs were used to create line profiles along the rod in the axial direction. Then, the mean and standard deviation of the recovery coefficient (RC) for the rods were calculated as follows:

20 $$\begin{aligned} RC\,=\,\frac{ Mean _{line}}{ Mean _{uniform}}, \end{aligned}$$

where $Mean_{line}$ represents the mean of the activity concentration for each line profile. The percentage standard deviation of the recovery coefficient for each rod was calculated as follows:

21 $$\% ST{D_{RC}}{\mkern 1mu} = {\mkern 1mu} 100{\mkern 1mu} \times {\mkern 1mu} \sqrt {{{\left( {\frac{{ST{D_{line}}}}{{Mea{n_{line}}}}} \right)}^2} + {{\left( {\frac{{ST{D_{uniform}}}}{{Mea{n_{uniform}}}}} \right)}^2}} .$$

#### Spill-over ratio

For the calculation of the spill-over ratio of water- and air-filled regions, two cylindrical VOIs with a diameter of 4 mm (half the physical diameter of the cylinders) and encompassing the central 7.5 mm in length were drawn in the water- and air-filled cylindrical inserts respectively. Then, the spill-over ratio (SOR) was calculated as follows:

22 $$\begin{aligned} SOR =\frac{ Mean _{ \scriptscriptstyle {VOI} }}{ Mean _{uniform}} \end{aligned}$$

where $Mean_{VOI}$ was the mean of the activity concentration in the water- or air-filled region VOI. The percentage standard deviation of SOR was calculated as follows:

23 $$\% STD_{SOR}\,=\,100\,\times \sqrt {{{\left( {\frac{{STD_{VOI}}}{{Mean_{VOI}}}} \right)}^2}\,+\,{{\left( {\frac{{STD_{uniform}}}{{Mean_{uniform}}}} \right)}^2}}.$$

#### Spatial resolution

The influence of different algorithms to the spatial resolution of the PET image was evaluated visually by the mini-Derenzo phantom study.

#### Percentage recovery signal and contrast-to-noise ratio

In the nude mice study, we compared the ability to preserve high-intensity features of different denoising approaches by two metrics. The first metric is the PRS in a VOI in the denoised image relative to noisy image, and the second is the CNR. These two metrics are important in clinical diagnosis and treatment monitoring. In comparison to PRS, which fails to capture the noise characteristics of the noisy images, CNR is more holistic, especially in studies where the goal is to detect potentially low remnant activity, the CNR can be quite critical.

Two VOIs were drawn for the calculation of PSR and CNR, a hot VOI in the lesion region and a low-intensity VOI that has a relatively uniform uptake in the background region (as shown in Fig. 12a). The PRS and CNR were calculated as follows:

24 $$\begin{aligned} PRS =\frac{M_{les}^{de}}{M_{les}^{un}}\, \times\,100\,\% \end{aligned}$$

and

25 $$\begin{aligned} CNR =\frac{|M_{les}-M_{back}|}{ STD _{back}} \end{aligned}$$

where $M_{les}^{de}$ and $M_{les}^{un}$ denote the mean intensity in the lesion VOI in the denoised images and noisy images respectively. $M_{les}$ denotes the mean intensity in the lesion VOI in denoised or noisy images, and $M_{back}$ and $STD_{back}$ represent the mean and standard deviation of intensity in the low intensity (background) VOI in the same images.

### Filtering parameters

In this work, 3D GF, BF, and ADF were applied. The only parameter of GF is the standard deviation of the Gaussian kernel, which is denoted as $\sigma _d$ and used to control the diffusion rate of the Gaussian kernel. For BF, there are two parameters to be determined to control the smoothing degree of the images, namely the geometric spread $\sigma _d$ and photometric spread $\sigma _r$, which determine the geometric closeness and photometric similarity of the neighboring voxels to be used in the smoothing process, in domain and range respectively. There are many flow functions which can be used in ADF, however, considering that one purpose of PET image denoising is to preserve a prominent signal in a homogeneous area, such as a focal lesion within a single anatomical region, we chose the exponential flow function in this work. The smoothing performance of ADF was controlled by two parameters, the diffusion rate $\lambda$, and the edge preserving parameter $\kappa$. The performance of the HOSVD-based algorithms depends on the setting of two patch-related parameters, patch size *p* and the number *K* of similar patches in a group; however, in a rational range, *K* would not significantly affect the denoising performance. Therefore *p* is the only free parameter which influences the accuracy in HOSVD-based algorithms.

**Table 1:** Filter parameters setting

GF	BF	ADF (%)	HOSVD-S
$\sigma _d$	$\sigma _r$	$\kappa$	p
0.5	25.5	60	5
0.8	*51.0*	65	*7*
*1.0*	76.5	70	8
1.5	112.0	*75*	10
2.0	127.5	80	13

In order to compare the performance of HOSVD-KSW with GF, BF, and ADF for PET image denoising, filtering parameters that make all these algorithms balanced should be determined; these selected parameters result similar standard deviation in the homogeneous regions in denoised images. In the phantom study, we chose this region as the uniform VOI, and in the mice study, the background VOI was used. The comparison between HOSVD-based algorithms was performed by using the same patch size *p*. For GF, the kernel size is fixed to five voxels. We fixed $\sigma _d$ at 4.5 in BF. For ADF, keeping $\lambda = 0.15$ ($\lambda \in [0,0.25]$ should be sufficiently small to make the diffusion process stable [18]) unchanged. The residual parameters were swept over the range listed in Table 1. Note that the parameter $\kappa$ in ADF was represented as the percentage of standard deviation inside the selected homogeneous region in this work.

**Fig. 4:**
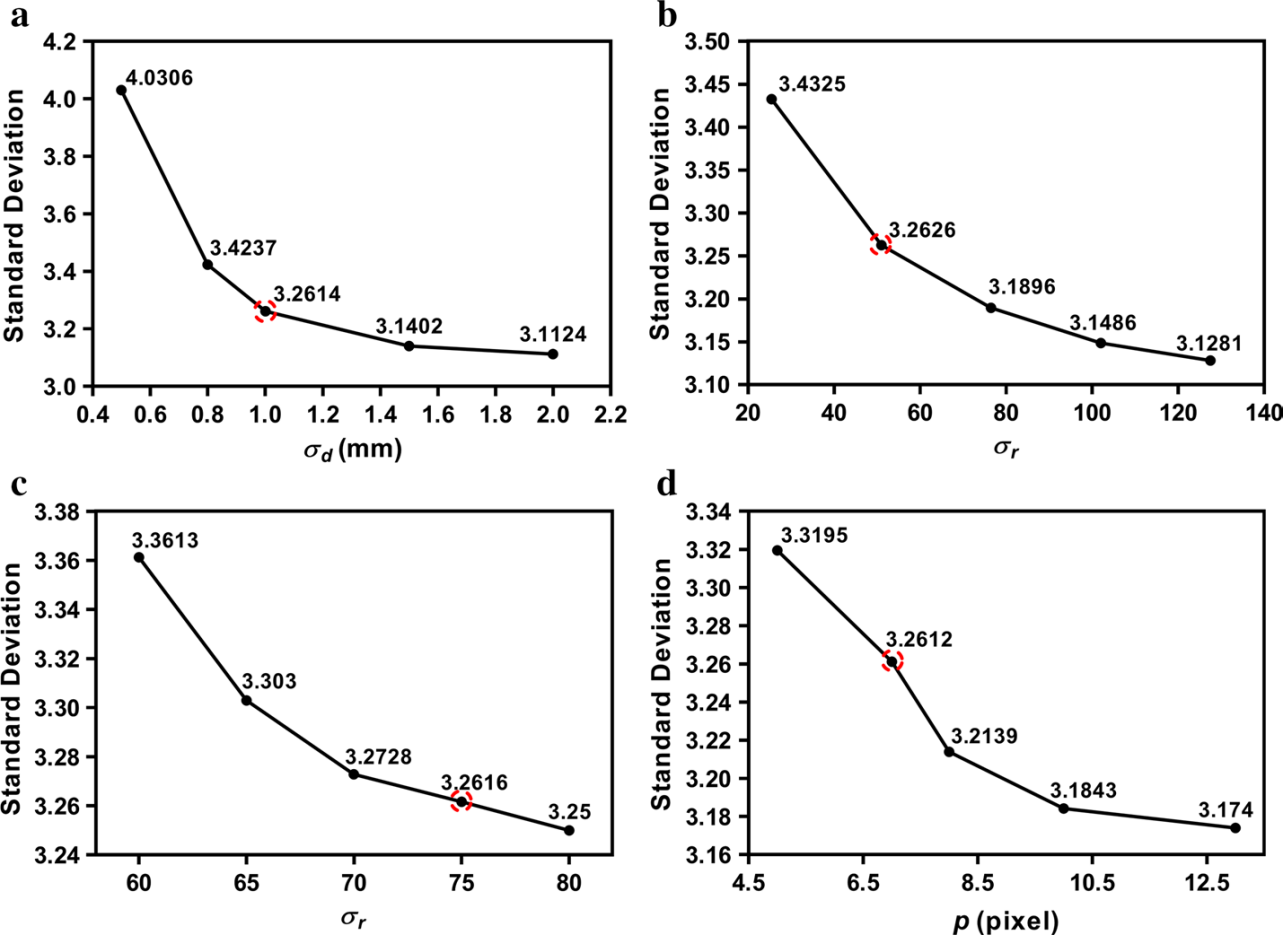
The standard deviation in a uniform VOI as a function of parameters in each filter in NEMA phantom study. **a** for Gaussian filter (GF), **b** for bilateral filter (BF) and **c** for anisotropic diffusion filter (ADF), and **d** for standard HOSVD-based filter (HOSVD-S)

Figure 4 shows the variation of the standard deviation in uniformity region VOI when different filtering parameters setting were used in the phantom study. The parameters corresponding to the standard deviation value circled in red dashed circles were chosen in the phantom study. For GF, $\sigma _d\,=\,1$ (1.07 mm FWHM) was used. For BF, $\sigma _d\,=\,4.5$ and $\sigma _r\,=\,25.5$. $\lambda\,=\,0.15$ and $\kappa\,=\,75\,\%$ for ADF. For HOSVD-S, HOSVD-W, and HOSVD-KSW, patch size was set to $p\,=\,7$.

For the nude mice study, the filtering parameters were $\sigma _d\,=\,1$ for GF, $\sigma _d\,=\,3.0$ and $\sigma _r\,=\,10$ for BF, $\lambda\,=\,0.15$ and $\kappa\,=\,80\,\%$ for ADF, and $p\,=\,9$ for HOSVD-S, HOSVD-W, and HOSVD-KSW.

## Results

### Image quality

**Fig. 5:**
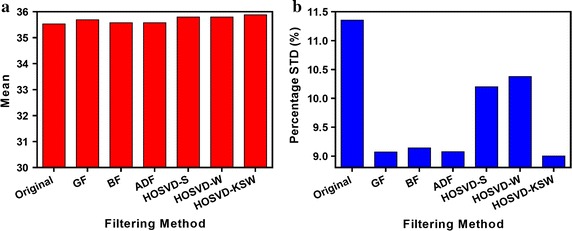
The mean and percentage standard deviation (PSR) of normalized counts in the uniform VOI in NEMA phantom study. **a** for the mean value, and **b** for the PSR

**Fig. 6:**
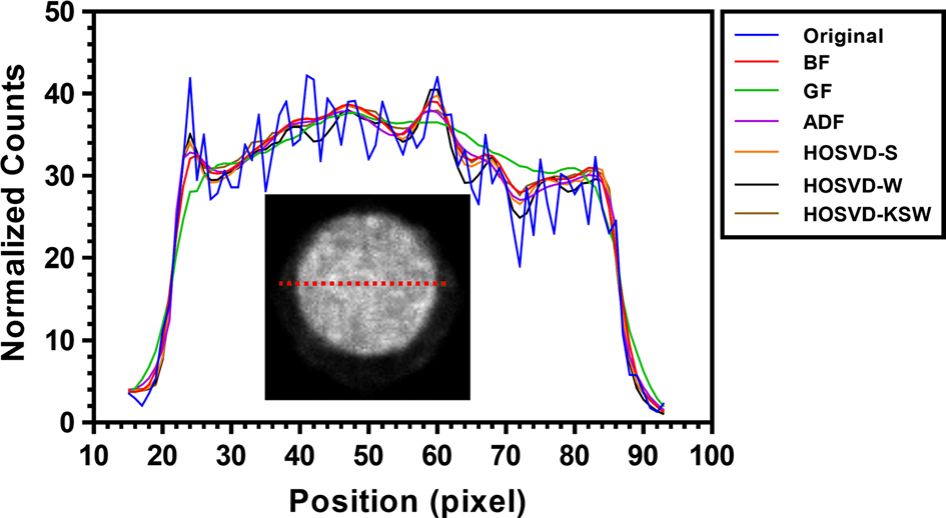
Counts profiles of the cross-section in uniform region in noisy and denoised images in NEMA phantom study

Figure 5 shows the mean (Fig. 5a) and percentage standard deviation (Fig. 5b) of the noisy and denoised image in the uniform VOI. From this result, all of these filters can preserve the mean and improve the smoothness in the uniform region. However, HOSVD-KSW yielded a similar smoothing result with GF, BF, and ADF, which were smoother than HOSVD-S and HOSVD-W. As was expected, GF yielded an over-smoothed boundary in the uniform region while BF and ADF preserved boundary. A similar edge-preserving result also could be observed in HOSVD-based methods. This can be seen in Fig. 6.

**Fig. 7:**
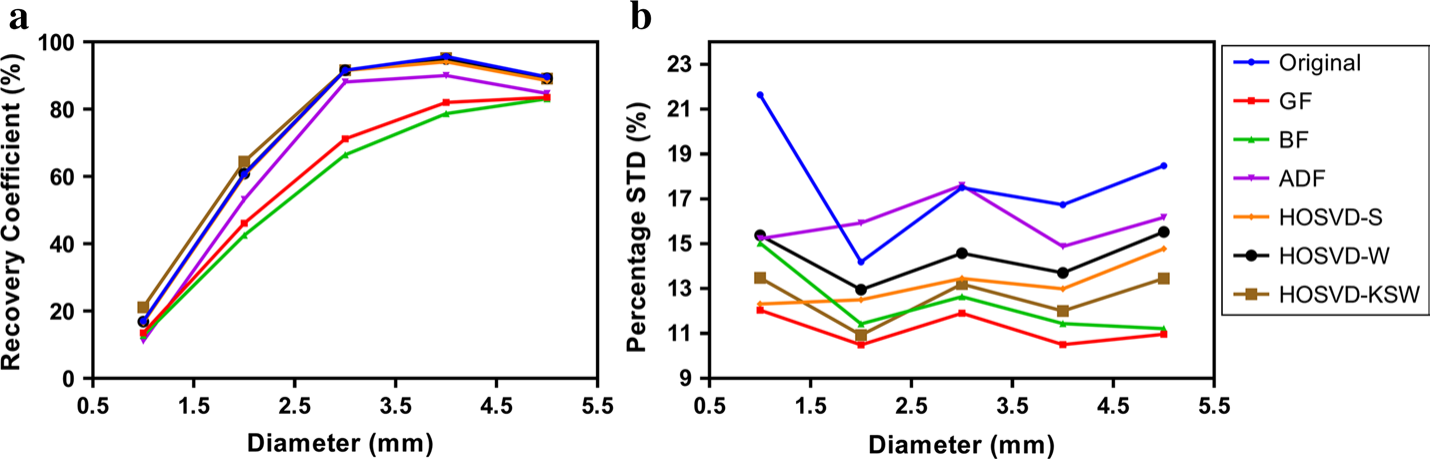
The recovery coefficient (RC) and percentage standard deviation (PSTD) of normalized counts in VOI within rod regions in NEMA phantom study. **a** for the recovery coefficient and **b** for the percentage standard deviation

Figure 7 shows how the recovery coefficients (RC) of rods varies with diameters in the noisy and denoised image under different algorithms. From Fig. 7a, the GF, BF, and ADF deteriorated the RC over all of the rods, while the HOSVD-based algorithms could preserve the RC, and the HOSVD-KSW improved RC slightly even with a smaller rod diameter. Figure 7b shows that HOSVD-S is less stable when compared with HOSVD-KSW, even though the Wiener filter step in HOSVD-W would improve this shortage in HOSVD-S.

**Fig. 8:**
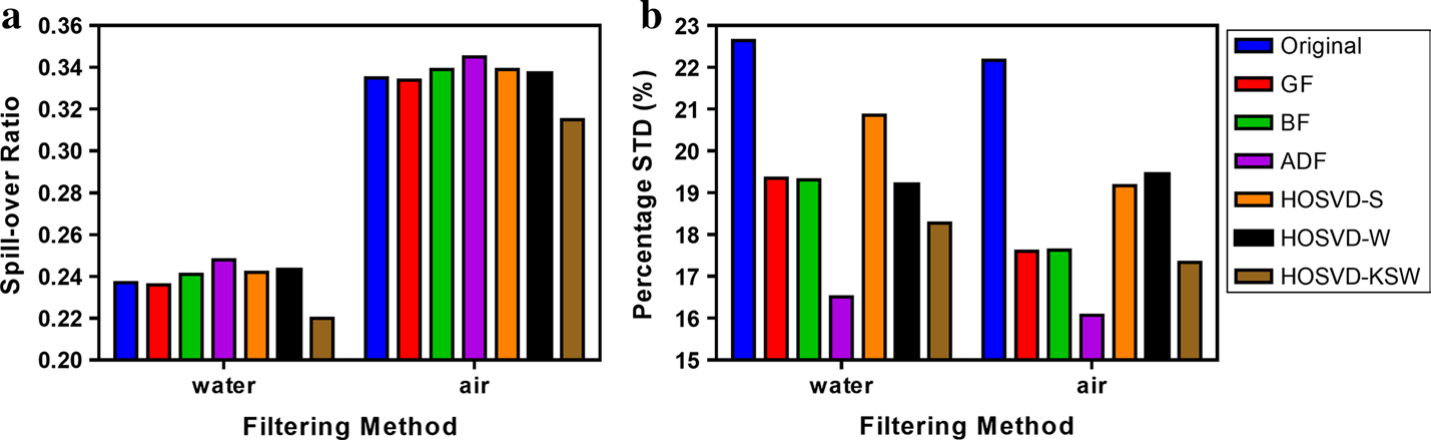
Results of spill-over ratio (**a**) and its percentage standard deviation (**b**) for the cavities filled with water and air in NEMA phantom study

**Fig. 9:**
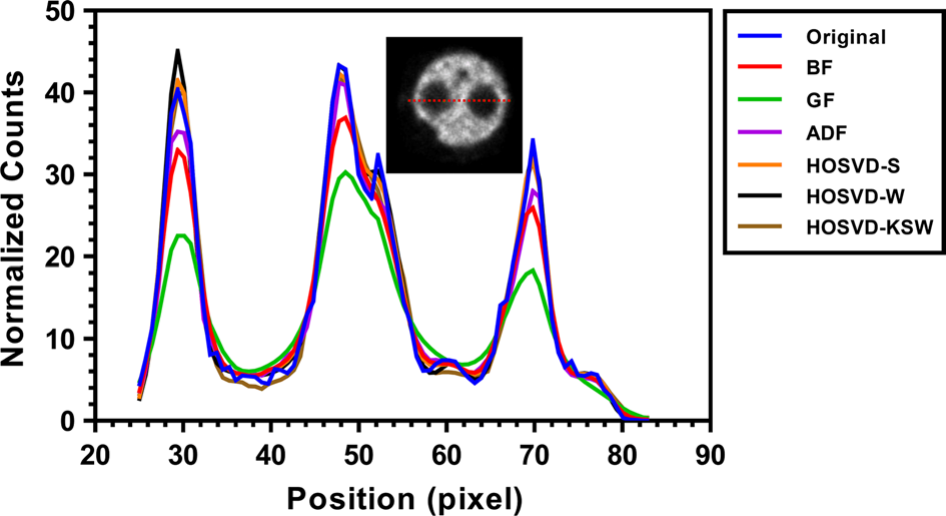
Counts profiles of the cross-section in water- and air-filled region in noisy and denoised images in the NEMA phantom study

Figure 8 shows the results of the spill-over ratio and the corresponding percentage standard deviation for the cavities filled with water and air. As shown in Fig. 8a, the HOSVD-KSW decreased SORs both in water- and air-filled regions while other compared methods enlarged it besides the GF. However, compared with HOSVD-KSW, the improvement of GF to SOR in water- and air-filled regions was negligible. Again, the percentage standard deviation (%STD) of SOR in water- and air-filled regions resulting from the HOSVD-KSW are comparable to those from GF and BF, and the HOSVD-S and HOSVD-W resulted in a higher %STD than other algorithms although both of them reduced it when compare to the noisy image, this was shown in Fig. 8b. Figure 9 shows the profiles of the dotted lines drawn in the center of the water/air chambers region, a reduction of the normalized counts in cold chambers could be observed in HOSVD-KSW.

### Mini-Derenzo phantom

**Fig. 10:**
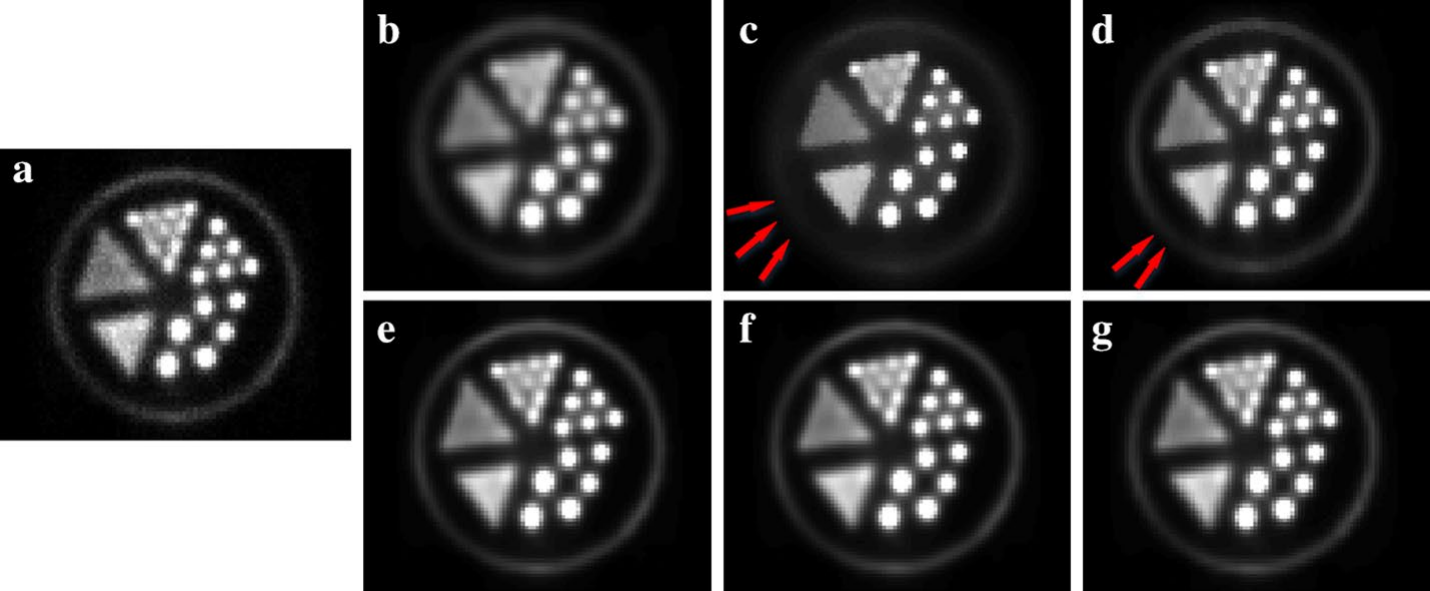
Transaxial slices that cut through the center of the mini-Derenzo phantom. **a** noisy image. **b** GF denoised image with FWHM = 1.07. **c** BF denoised image with $\sigma _d = 4.5$ mm, $\sigma _r = 1.5$. **d** ADF with $\lambda$ = 0.15, $\kappa$ = 4. **e** HOSVD-S, **f** HOSVD-W, **g** HOSVD-KSW with *p* = 9

The results of the mini-Derenzo phantom study are shown in Fig. 10. The noisy image is shown in Fig. 10a. The denoised image using GF with $\sigma _d=1$ is shown in Fig. 10b. As expected, the GF smoothed out signal and noise simultaneously. The BF denoised image demonstrated better preserved hot boundaries than the GF, but the cold boundaries were smoothed out, as denoted by the red arrow in Fig. 10c. ADF yielded a result between GF and BF as shown in Fig. 10d. HOSVD-based algorithms demonstrated better-preserved results of cold boundaries and hot regions. There is no evident difference in the denoised images resulting from HOSVD-S, HOSVD-W, and HOSVD-KSW. This may have benefit from the accurate structural information existing in the mini-Derenzo phantom, as the simple similarity measure used in HOSVD-S is just enough for similar patch selection for a certain reference patch.

As shown in Fig. 10, the activity-filled area with the diameter of 1.6 mm can be distinguished clearly in all denoised images. However, the area with a diameter of 1.2 mm was smoothed in GF, BF, and ADF, while the HOSVD-based algorithms demonstrated better-preserved details in this area. It is clear that the HOSVD-based algorithms can preserve the spatial resolution of the PET system well.

### Nude mice study result

**Fig. 11:**
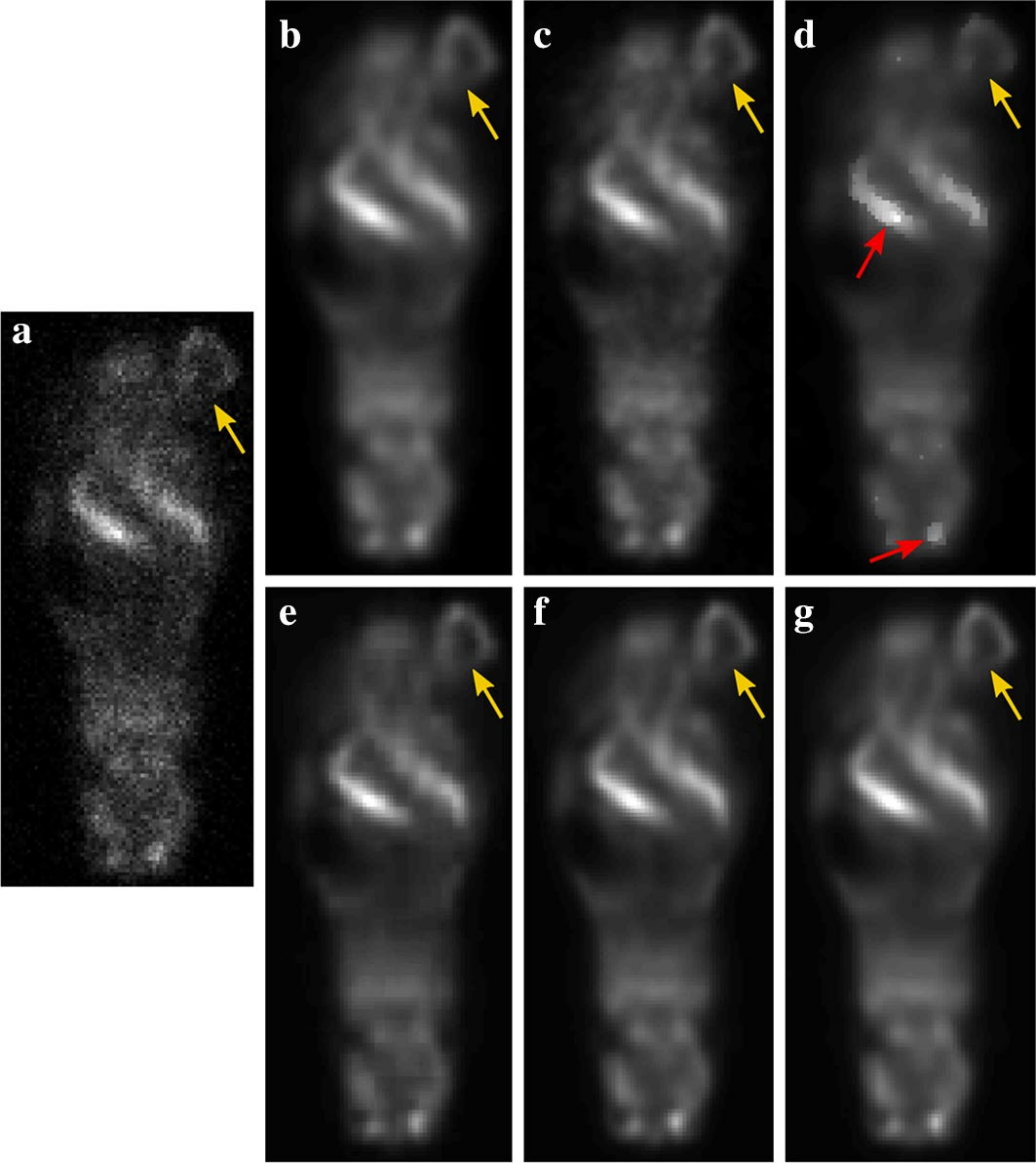
Transaxial slices that cut through the lesions in the mice study. **a** noisy image. Images filtered with GF (**b**) at FWHM = 1.07, BF (**c**) at $\sigma _d$ = 3.0, $\sigma _r$ = 10 and ADF (**d**) at $\lambda$ = 0.15, $\kappa$ = 2. **e**–**g** are the denoised images with HOSVD-S, and HOSVD-W and HOSVD-KSW respectively, with the patch size of *p* = 9

**Fig. 12:**
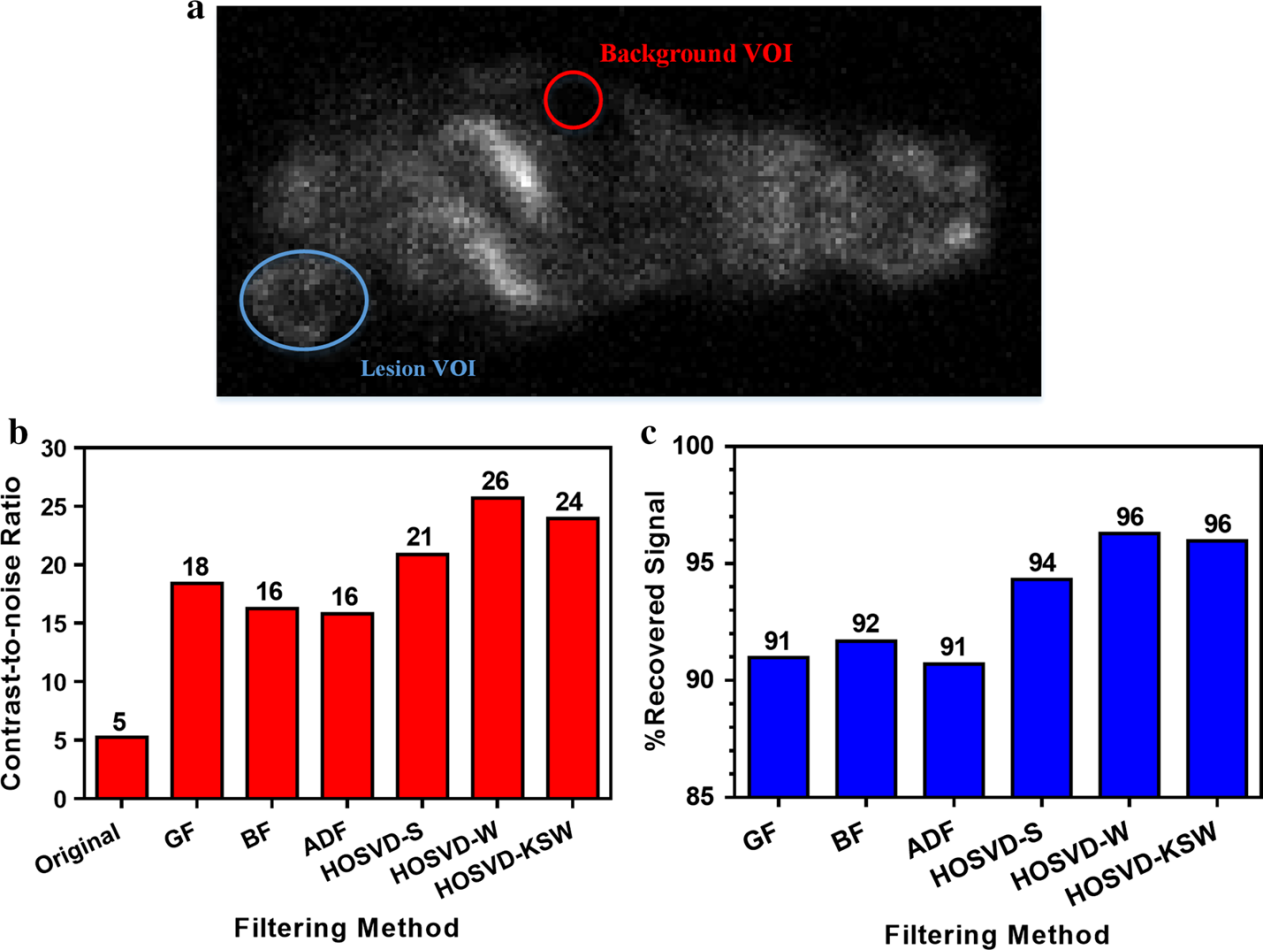
Calculation results of contrast-to-noise ratio (CNR) and percentage recovered signal (PRS) in mice study. The VOIs are shown in **a**, where the background VOI is marked with a *red circle* and the lesion VOI with *light blue circle*. The CNR in noisy and denoised images are shown in **b**, and the PRS in images with different filters are shown in **c**

Figure 11 presents coronal slices through lesions in the nude mice 18F-FDG PET study. PET image shown in (a) is a noisy reconstructed image, (b), (c), (d), (e), (f), and (g) are the images that are denoised by GF, BF, ADF, HOSVD-S, HOSVD-W, and HOSVD-KSW, respectively. Lesions are denoted by yellow arrows in these images. The lesion is ellipsoidal with its length in three axis are  1.1,  0.9 and  0.6 cm respectively, and the volume is  2.5 mL. It can be observed that GF suppressed noise as well as the lesion and organ boundaries. BF suppressed noise to a similar extent as GF in the background region, and yielded sharper boundaries of the lesion and organs, as shown in Fig. 11c. ADF preserved the high contrast objects, but low contrast details such as the lesion were smoothed, and there were stair-step artifacts in the boundaries of high contrast objects as denoted by red arrows shown in Fig. 11d. All of the HOSVD-based algorithms suppressed noise effectively, however, HOSVD-S yielded stair-step artifacts, as shown in Fig. 11e. Both HOSVD-W and HOSVD-KSW preserved the boundaries of the lesion and other high contrast objects, as can be seen in Fig. 11f, g.

Quantitative results from the nude mice 18F-FDG PET study are shown in Fig. 12. VOIs in the lesion and background regions used for computing the CNR and PSR are delineated in Fig. 12a. It can be seen that all compared algorithms improved the CNR (Fig. 12b) in the lesion region in comparison to the noisy image. However, GF, BF, and ADF yielded worse results than HOSVD-based algorithms. GF improved CNR by 249 % (CNR = 18) compared to the noisy image (CNR = 5), and introduced 9 % (PSR = 91 %) reduction in average normalized counts in the lesion. BF and ADF improved CNR by 208 and 200 %, and yielded 8 % (PRS = 92 %) and 9 % (PNS = 91 %) reduction in the average normalized counts in the same region, respectively. These demonstrated that traditional edge preserving filters could not achieve better quantitative performance than GF when their smoothness of the background region was similar. However, HOSVD-based algorithms introduced a better CNR and yielded better PRS than other compared algorithms, among which HOSVD-W yielded the best performance, with a 387 % increase in CNR, while maintaining similar average normalized counts (PSR = 96 %) in the lesion when compare to the other two HOSVD-based methods. Compared to HOSVD-W, HOSVD-KSW yielded the second best CNR and PRS with CNR = 24 and PSR = 96 %, but its time consumption was dramatically lower than for the HOSVD-W.

## Discussion

In this study, we investigated the use of a novel denoising algorithm based on high-order singular value decomposition in PET image denoising. This algorithm performs image denoising by exploiting the sparsity between similar patches by using the HOSVD transform, and offers a simple and elegant method for handling sparsity among similar patches. The HOSVD bases are learned from the image and thus are more adaptable to the image content and may achieve a much sparser representation than fixed bases such as wavelet and discrete cosine bases.

To evaluate the performance of HOSVD-based denoising algorithms in PET image filtering, we performed three 18F-FDG PET studies with: a NEMA small animal image quality phantom, a mini-Derenzo phantom, and a nude mice with 4T1 tumors. The noisy reconstructed PET images were filtered by GF, BF, ADF, and HOSVD-based denoising methods. Our results demonstrated that HOSVD-based methods yielded consistently superior performance compared with GF, BF, and ADF, in terms of visual quality and quantitative metrics. HOSVD-W and HOSVD-KSW recovered the small hot region better, while preserving the spatial resolution and reducing the SOR of the background regions. On condition that the smoothness of the background region is similar with each other, both methods suppressed the noise in soft tissues effectively and preserved the organ boundaries.

The straight usage of HOSVD-S might lead to stair-step artifacts in denoised PET images. This is because the patch similarity measure may collect patch pairs with quite different structures in the Anscombe transformed image with identical weight. If these patches are grouped into a 4D stack then the HOSVD bases that learned from them are not accurate, and lead to inappropriate information spreading into the final denoised PET image. While the Wiener filter step used in HOSVD-W can modify this shortcoming, the time consumption will dramatically increase to about twice as long as the HOSVD-S. Our results demonstrated that the proposed method HOSVD-KSW yielded similar performance compared with HOSVD-W, and the time-consumption was similar with HOSVD-S.

The good denoising performance of the HOSVD-KSW is attributed to two main features. The first feature is that HOSVD-KSW groups the similar patches into a stack with a weighted manner, where the weight is calculated according to the similarity between reference patch and special patch in domain and statistics. This will make the HOSVD bases more accurate than those in HOSVD-S, and thus remove the stair-step artifacts existing in HOSVD-S denoised images. The second feature is that the similarity weight of similar patches is calculated by considering two factors. The first factor is the Euclidean distance between a similar patch and reference patch from the similarity measure process, and the second factor is the *p* value output of the K-S test of the difference patch between the reference patch and the similar patch. This two-part criterion enables the high accuracy of the HOSVD bases.

The main shortcoming of the proposed HOSVD-KSW method for 3D PET image denoising is that we considered the noise in the reconstructed PET images as the Poisson distribution. It was not accurate enough, as well known, and a more accurate noise model and corresponding variance-stabilizing transformation should be used to improve the reliability of the results.

## Conclusions

In summary, our study demonstrated that the HOSVD-W and the proposed HOSVD-KSW methods yield improved image quality while preserving the accuracy of lesion quantification. Considering that the time-consumption of the HOSVD-KSW is about half of the HOSVD-W, we believe that the HOSVD-KSW is more practical than the HOSVD-W at a very low performance compromise.

## Additional file

10.1186/s12938-016-0221-y Original 3D PET images data used in this work to generate the results.

## Abbreviations

PET: positron emission tomography
HOSVD: higher-order singular value decomposition
AVST: anscombe variance-stabilizing transformation
MLEM: maximum likelihood expectation maximization
OSEM: ordered subset expectation maximization
GF: Gaussian filter
BF: bilateral filter
ADF: anisotropic diffusion filters
NLM: nonlocal mean
BM3D: block-matching three dimension filter
SNR: signal-to-noise ratio
SVD: singular value decomposition
PRS: percentage recovered signal
VOI: volume of interest
CNR: contrast-to-noise ratio
HOSVDS: standard HOSVD
HOSVD-W: wiener filter-augmented HOSVD
K-S test: Kolmogorov-Smirnov test
HOSVD-KSW: weighted HOSVD
PSF: point spread function
STD: standard deviation
ROI: region of interest
RC: recovery coefficient
SOR: spill-over ratio
